# Identifying Opportunities for Strategic Policy Design to Address the Double Burden of Malnutrition through Healthier Retail Food: Protocol for South East Asia Obesogenic Food Environment (SEAOFE) Study

**DOI:** 10.3390/ijerph19010528

**Published:** 2022-01-04

**Authors:** Sirinya Phulkerd, Cut Novianti Rachmi, Mohd Jamil Sameeha, Elaine Q. Borazon, Anne-Marie Thow, Helen Trevena, Adila Fahmida Saptari, Yong Kang Cheah, Che Aniza Che Wel, Vanessa T. Marquez, Teeranong Sakulsri, Natjera Thongcharoenchupong, Bee Koon Poh

**Affiliations:** 1 Institute for Population and Social Research, Mahidol University, Nakhon Pathom 73170, Thailand; sirinya.phu@mahidol.edu (S.P.); teeranong.sak@mahidol.edu (T.S.); birt_redmachine@hotmail.com (N.T.); 2 Reconstra Utama Integra, Jakarta 12150, Indonesia; cutnovianti@gmail.com (C.N.R.); adilafsaptari@gmail.com (A.F.S.); 3 Centre for Community Health Studies (ReaCH), Faculty of Health Sciences, Universiti Kebangsaan Malaysia, Kuala Lumpur 50300, Malaysia; sameeha@ukm.edu.my; 4 International Graduate Program of Education and Human Development (IGPEHD), College of Social Sciences, National Sun Yat-Sen University, Kaohsiung City 80424, Taiwan; elaineqborazon@mail.nsysu.edu.tw; 5 Menzies Centre for Health Policy and Economics, University of Sydney, Sydney 2006, Australia; annemarie.thow@sydney.edu.au (A.-M.T.); helen.trevena@sydney.edu.au (H.T.); 6 School of Economics, Finance and Banking, Universiti Utara Malaysia, Sintok 06010, Malaysia; yong@uum.edu.my; 7 Faculty of Economics and Management, Universiti Kebangsaan Malaysia (UKM), Bangi 43600, Malaysia; aniza@ukm.edu.my; 8 School of Economics, University of the Philippines-Diliman, Quezon City 1101, Philippines; vtmarquez@up.edu.ph

**Keywords:** malnutrition, food retail, obesogenic environment, policy, South East Asia

## Abstract

Effective policies that address both the supply and demand dimensions of access to affordable, healthy foods are required for tackling malnutrition in South East Asia. This paper presents the Protocol for the South East Asia Obesogenic Food Environment (SEAOFE) study, which is designed to analyze the retail food environment, consumers’ and retailers’ perspectives regarding the retail food environment, and existing policies influencing food retail in four countries in South East Asia in order to develop evidence-informed policy recommendations. This study was designed as a mixed-methods sequential explanatory approach. The country sites are Malaysia, Indonesia, the Philippines, and Thailand. The proposed study consists of four phases. Phase One describes the characteristics of the current retail food environment using literature and data review. Phase Two interprets consumer experience in the retail food environment in selected urban poor communities using a consumer-intercept survey. This phase also assesses the retail food environment by adapting an in-store audit tool previously validated in higher-income countries. Phase Three identifies factors influencing food retailer decisions, perceptions, and attitudes toward food retail policies using semi-structured interviews with selected retailers. Phase Four recommends changes in the retail food environment using policy analysis and semi-structured interviews with key stakeholders. For the analysis of the quantitative data, descriptive statistics and multiple regression will be used, and thematic analysis will be used to process the qualitative data. This study will engage stakeholders throughout the research process to ensure that the design and methods used are sensitive to the local context.

## 1. Introduction

Countries in South East Asia (SEA) are experiencing all forms of malnutrition due to a rapid dietary transition that is occurring throughout the region [1]. The Global Nutrition Report for South East Asia shows that, although prevalence of stunting has decreased, it remains high (38.5% to 24.7%), while the prevalence of overweight has more than doubled (3.2% to 7.5%) between 2000 and 2019 among children aged below 5 years [2]. This double burden of malnutrition, which refers to the coexistence of under- and over-nutrition in the population, occurs not just among children but also in other age groups [3]. Among adults, the prevalence of underweight is on the decline, whereas overweight and obesity rates have increased rapidly in the last two decades. Micronutrient deficiencies are also prevalent, whereby 34–48% of children had vitamin D insufficiency [4], 12–18% had iron deficiency and 28–60% had zinc deficiency [5]. Moreover, in the last decade, anemia among women of reproductive age has worsened, from a moderate level to a severe public health problem, particularly among pregnant women (35.9% in 2008 to 40.3% in 2016). The economic and demographic transition (i.e., urbanization) and the obesogenic environment are drivers of the increasing prevalence of obesity and diet-related non-communicable diseases (NCDs) [6]. The term “*obesogenic environment*” refers to “*an environment that promotes weight gain and one that is not conducive to weight loss*” [7]. Despite this growing burden, policy has failed to shift from a focus on access to calories to improving access to foods of higher nutritional quality and reducing access to, and marketing of unhealthy foods.

SEA countries have experienced an increase in overweight and obesity prevalence, including Indonesia, Malaysia, the Philippines, and Thailand. In Indonesia, the prevalence of overweight and obesity in 2013 was 13.5% and 15.4%, respectively. In the span of five years, the prevalence of overweight increased by 0.1 percentage points, and obesity increased by 6.4 percentage points [8,9]. In Malaysia, between 2015 and 2019, the prevalence of overweight for males decreased by 0.8 percentage points to 30.8%, while the prevalence of obesity in males increased slightly by 0.3 percentage points to 15.3% [10]. During the same period, the prevalence of overweight among Malaysian females increased by 1.7 percentage points to 30.0%, while the prevalence of obesity in females increased slightly by 4.1 percentage points to 24.7%. In the Philippines, the prevalence of obesity, overweight, and diabetes all increased from 2015 to 2018 [11]. The prevalence of overweight increased by 4.1 percentage points, while obesity increased by 2.4 percentage points. In Thailand, between the years of 2008/9 and 2013/4, the prevalence of overweight/obesity increased in both males and females. The proportion of females who were clinically overweight/obese was higher than that of their male counterparts in both time periods [12,13].

Modifying the obesogenic environment at the community and policy level to promote healthy food should help to create and reinforce social norms for the consumption of healthier food [14]. As part of a comprehensive approach to effectively address the coexistence of both under- and over-nutrition in SEA, there is a need to identify specific, contextually appropriate and effective policies that address both the supply and demand of access to and promotion of healthy and unhealthy foods. 

ASEAN (Association of South East Asian Nations) member governments have expressed a high-level commitment to combat all forms of malnutrition, which is believed to be slowing down socio-economic development [15]. However, the capacity of a country to reduce malnutrition is limited by an incomplete understanding of contextual and policy factors that are drivers of the food retail sector. There are often sub-national dynamics, which may shape the food environment in undesirable ways, and thereby undermine efforts of national government. These dynamics include, for example, decentralization, orderliness, and anti-informality in public health. The private sector also plays an important role in shaping the policy process. Previous partnerships between the food industry and government have been strongly criticized for lacking effectiveness and transparency, and having an over-riding focus on reputational enhancement rather than public health [16].

Many questions remain unanswered regarding the retail food environment in SEA. For example, it is unclear to public health policymakers what the retail food landscape looks like, how the average consumer interacts with the retail food environment, what the drivers of food retailer decision-making are, and what public policies are of most relevance to food retail, and why. In addition, the growing disruptions caused by the COVID-19 pandemic continue to present challenges to supply and demand of consumable items. The pandemic has exposed the fragility of the food system, particularly food distribution [17], which is underpinned by economic and power relations. The resulting imbalance may have a lasting impact on household food dynamics, consumer food experience, food availability, and food affordability. These phenomena could lead to the reshaping of food retail systems [18,19] and, as such, may impact on diet inequality [20].

To increase access to healthy foods, retail stores can play an important role in driving change by offering consumers a wider choice of culturally acceptable and affordable foods to promote healthy eating. In common with many nations, SEA countries are currently undergoing a food retail transition. For example, Thailand and Malaysia have experienced a rapid growth in large food retail formats, such as supermarkets [21,22]. Meanwhile, a decline in traditional retail markets with a shift to more digital (i.e., online) shopping is also observed. This has resulted in a loss of fresh, healthy, affordable food for lower-income Thais, and a dilution of regional variation in culinary culture [23]. Modern food retail formats are rapidly becoming the main community food source for many populations in the region, much in the same way that central food distribution systems operate in more economically advanced countries [24]. For example, supermarkets and convenience stores now provide the majority of energy for the US population [25]. Supermarkets are the dominant shopping venue in Australia; however, less than half of supermarket packaged foods are considered ‘healthy’ [26].

In addition, packaged and processed foods are increasingly available in convenience stores across the SEA region. Use of convenience stores is growing rapidly, with sales in 2020 at least double those in 2015 in the Philippines, Malaysia, and Indonesia [27]. However, there is a paucity of evidence explaining the retail food environment, consumer purchases, and food retailer practices in SEA countries. In addition, there is a dearth of analysis of policy opportunities and challenges in creating healthy retail food environments [28]. The SEA Obesogenic Food Environment (SEAOFE) study was conceived to fill this gap. 

The aim of the SEAOFE study is to improve understanding of the retail food environment, consumers’ and retailers’ perspectives on factors influencing their food retail-related decisions, as well as the existing national-level policies and actions influencing food retail in SEA. The results of this research should help to inform policy design and implementation across the sectors that govern the food supply. The specific objectives of the research are as follows:

1. To analyze the characteristics of the national food retail landscape in Malaysia, Indonesia, the Philippines, and Thailand.
2. To explore the consumer experience of their retail food environment, and to assess the retail food environment in selected urban poor areas.
3. To analyze retailer decision-making relevant to selling healthier food in urban poor areas.
4. To analyze the policy landscape relevant to the national retail food environment.

## 2. Materials and Methods

**Figure 1:**
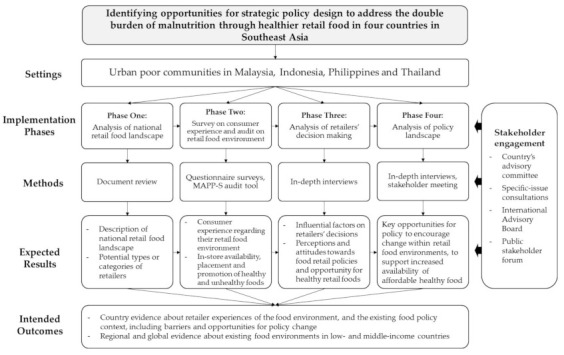
Schematic presentation of the SEAOFE study Protocol.

The SEAOFE uses a mixed-methods sequential explanatory study design, and the proposed implementation consists of four phases (Figure 1). The research integrates approaches from public health nutrition, business, and political science to assess the multidisciplinary challenges of food retail policy for improving nutrition. The study will engage a wide range of stakeholder groups (e.g., policymakers, retailers, academics, civil society) through country-level advisory committees and specific-issue consultations with country experts. An international advisory group for the study consists of members with expertise in food retail, food policy, population nutrition, and research methods. The advisory group is scheduled to meet twice a year to provide strategic advice through sharing of knowledge and experience in the retail food environment and policy research.

This study defines the ‘retail food environment’ according to the International Network for Food and Obesity/NCDs Research, Monitoring and Action Support (INFORMAS) [29], which can be divided into the ‘community food environment’ (the type, availability and accessibility of retail food outlets) and the ‘consumer food environment’ (the availability, prices, promotions, and nutritional quality of products available within stores). INFORMAS is a global network of public-interest organizations and researchers, aiming to monitor, benchmark, and support public and private sector actions to create a healthy food environment and reduce obesity, NCDs and their related inequalities [30].

The SEAOFE study will be conducted in Malaysia, Indonesia, the Philippines, and Thailand. These countries were selected based on similarities and differences in relation to food and nutrition policy, as well as socio-demographic and economic disparities. These countries are all ASEAN member nations with middle-income economies [31], and vary in population size (Indonesia: 261 million, the Philippines: 103 million, Thailand: 68.9 million, and Malaysia: 31.2 million) and GDP (highest to lowest: Malaysia, Thailand, Indonesia, and the Philippines) [32]. While they all experience the double burden of malnutrition [2], there are differences in the prevalence of NCDs, overweight, and obesity. They have all experienced a continuous trend toward urbanization, with the fastest rate in Malaysia, followed by Indonesia, Thailand, and the Philippines, respectively [33].

This study will be conducted in line with appropriate standards of research ethics, and approval of the study protocol has been obtained from the research ethics authority in each of the study countries. Reports of findings will present aggregated anonymous data in all publications. 

The proposed study will be conducted over four years, with approximately one year planned for each phase. In September 2020, the first Coordination Meeting among the four study countries was convened to discuss the protocol development. 

### 2.1. Definition and Scope

“Retailing,” based on the definition of the International Standard Industrial Classification (ISIC), is the “*resale (sale without transformation) of new and used goods, mainly to the general public for personal or household consumption or utilization, by shops, department stores, stalls, mail-order houses, hawkers, peddlers, consumer cooperatives, auction houses, etc. Most retailers take title to the goods they sell, but some act as agents for a principal, and sell either on consignment or on a commission basis*” [34]. The retail sector sells food and non-food items requiring minimal or no additional processing before selling directly to the consumers [35]. Sale without transformation includes operations related to trade (e.g., sorting, grading, assembling, mixing, bottling, packing, downsizing, storage, cleaning, and drying of raw product) [34]. Based on these definitions, the researchers define “food retailers” as those engaged in the resale (sale without transformation) of food and beverage items, generally for off-site consumption. These outlets may have in-store meat packaging facilities, in-store bakeries, and/or ready-made food for individual or institutional consumers (e.g., kiosks, restaurants, hotels) [35]. For the purpose of this research, these outlets do not include food service establishments “*where food is prepared and intended for individual portion service and includes the site at which the individual portions are provided, whether consumption occurs on or off the premises*” [36].

This study will focus on the following major categories of food retailers, as defined by FAO, 2009 [35]:

Hypermarket: A store with a sales area of over 2500 m^2^ with at least 35% of selling space devoted to non-food items. Sometimes, hypermarkets are also called “*super stores,*” which are a combination of a supermarket and a department store. Hypermarkets are usually located in suburbs due to the limited space available in city centers, and the need for large parking areas for shoppers. Some hypermarkets are located close to residential areas and can be adjacent to shopping centers that sell consumer electronics, furniture, durable and leisure goods, etc.).

Supermarket: A store with a sales area of typically 400 to 2500 m^2^ with at least 70% of its selling space dedicated to food and beverage products.

Convenience stores (C-stores): These outlets sell a wide range of goods, and have extended operating hours. A convenience store is often located alongside a busy road, as part of a gas or railway station, or in a densely-populated urban area. Product selection is limited compared with supermarkets and, in many stores, only one or two choices are available for each type of product. Prices in convenience stores are typically higher than in supermarkets.

Modern retailers rely on formal markets, are easily accessible, and have a high level of food promotion strategies [37]. Moreover, these modern food retailers can be classified in terms of outlet size (micro, small, medium, large) on the basis of asset volume, number of employees, and sales turnover. Definitions vary across the four study countries. “*Traditional retailers*” are those who source merchandise from traditional traders (who buy from smallholder farmers) and sell to consumers in wet markets (e.g., daily village kiosks, daily side-of-the-road traditional markets, daily/weekly/regional traditional markets), or modern retailers or those who procure mainly from domestic and multinational food manufacturers and sell through modern supermarket outlets [38]. 

### 2.2. Phase One: Food Retail Landscape

This phase will describe and interpret the characteristics of the retail food environment in each study country, and provide the basis for Phase Two selection of an important retail sector for in-depth analysis. Findings from an interdisciplinary review of recent data and relevant literature will be used to define the characteristics of the current retail food environment in each study country. A broad range of databases will be searched. Source materials that are available in either English or local language(s) will be included. Next, the researchers will identify suitable locations and/or categories of retailers for in-depth focus in the subsequent phases. This step will use the analytic hierarchy process (AHP) for prioritization of study sites.

#### 2.2.1. Data Collection

The first phase will initially use Google to search for sources of data related to the national food landscape for each country, and limit the search to the first ten screen-pages of results. In addition, the researchers will scan books, reports, working papers, websites of food retailers/government/intergovernmental agencies, market research databases (Euromonitor International), and other grey literature for contextual information of relevance to the retail food environment. Each country will use the same Microsoft Excel spreadsheet format to map their findings for each indicator from several data sources as identified by the Google search. This phase aims to comprehensively understand the recent dynamics of the retail food environment, especially in the context of the response to the COVID-19. Therefore, data sources will be limited to reports published/completed between 2010 and 2020. Researchers from each country team will carry out the search for their country, and will translate relevant data labels/explanations into English if needed.

Similarly, the researchers will collect information about the spread of COVID-19 in each study country to explore the potential implications of the pandemic for food retail. Each research team will use the same keywords to search for relevant news items/articles, which describe the impact of COVID-19 on the retail food environment in their respective country. News text on food retail will be obtained through Internet search, Scopus, and other relevant news outlets. Throughout this phase, the researchers define ‘food retail’ as an outlet that sells fresh and packaged food or food products, excluding food service (i.e., in-store dining or take-out of prepared meals/beverages).

For the selection of news source, the research teams will use those most relevant to their country. The search will be limited to the major news outlets in each country. Each country will then collect all the news items/articles that appear in the first five screen-pages of the keyword search. All items will be summarized and coded per theme. At least two coders will confirm each other’s assignment of an item to a particular code.

The research teams will determine the timeframe for the search according to each country’s COVID-19 situation. The beginning of the timeframe should start on the week that their respective government enacted any national regulation or measure to contain the spread of COVID-19, and extend through the end of 2020.

Data will then be entered into a pre-formatted Excel spreadsheet to allow further analysis within and between countries. All data sources, by year published and the name of the individual who conducted the search, will be documented on the first page of the spreadsheet.

#### 2.2.2. Data Analysis

To analyze and synthesize the findings from the included literature, this phase will employ a ‘narrative synthesis,’ which is a flexible approach that allows the reviewers to be reflective and critical when reporting on the studies identified in this phase [39].

Two key outputs of this phase will be the provision of comparable data to classify potential ‘types’ or ‘categories’ of retailers that could be considered ‘hotspots’ in the emerging obesogenic food environment of each study country. Each research team will use Phase One data to apply AHP [40,41] to narrow down the study locations and types of retailers to focus on in the next phases. 

In the AHP process, the research team in each country will invite three to seven experts in food retail and other relevant stakeholders, who are purposively selected, to rank the type of retailers by importance and relevance. The research teams will then prioritize food retailers based on the AHP method formula. The outcome of the AHP will determine what type/category of food retailer will be the focus of the subsequent phases of this study.

### 2.3. Phase Two: Consumer Experience and In-Store Food Environment

This phase will interpret the consumer experience of the retail food environment in selected urban poor areas. This will be conducted in relation to the retailer types identified in Phase One as having relevance to the obesogenic food environment for each study country. 

#### 2.3.1. Consumer Intercept Survey

##### Study Design, Setting and Population

This phase uses a cross-sectional design to assess consumer perception of their retail food environment and consumer behaviors in relation to this environment, and the association of these with socio-demographic characteristics (gender, age, weight, height, ethnicity, employment status, marital status, number of persons in household, household monthly income, and average expenditure on groceries per month) of the study population. The analysis will draw on concepts of food and nutrition environments and consumer behavior [30,42,43]. Concepts include the availability and adequacy of food, which are factors that can influence food environmental outcomes, and consumer demand.

The association of malnutrition with urban poverty is evident, especially in middle- and lower-income countries [44,45,46,47,48] and, therefore, this phase will purposively select urban poor areas in the capital city of each study country as the research sites, taking into account population density, urban poor population, and socio-economic characteristics of neighborhoods. In countries where population and socio-economic characteristics cannot be obtained, urban poor location will be obtained using AHP as described in Phase One. Using the output from Phase One, the researchers will determine retailer types in two countries (Indonesia and Malaysia), and the retailer type and urban poor location in the other two countries (Thailand and the Philippines). The eligible retail stores in the city will then be identified using GPS coordinates, and mapped using the Geocoding method (QGIS version 13.4). The eventual choice of retailers to study will be based on the proximity of residential buildings and retailers), ideally within a 1–4 km radius based on previous studies [49,50] or fastest travel time to reach a retail food outlet (5–10 min drive or 10–15 min walk [49,51]). Approximately 10% of the total retailers in the area will be selected as the study retailers.

Proportionate stratified sampling will be used to select respondents (aged 18 years or older) who report purchasing a food/beverage from the selected retailer in each study location. The respondent who shops at the selected retail outlet will be systematically intercepted (every nth person to avoid bias and ensure representativeness) based on pedestrian traffic. The minimum sample size for problem identification in marketing research is 500 respondents, while a typical sample size is 1000–2500 respondents [52]. Thus, this phase will recruit 1000 respondents per study country.

##### Data Collection

The SEAOFE questionnaire will be completed through an intercept survey using in-person or telephone interviews, or through an online survey platform, depending on the COVID-19 epidemic situation and restrictions in each country. The questionnaire will take approximately 15–30 min to complete, and consists of questions on sociodemographic characteristics, food shopping preference, factors influencing food purchase and the respondent’s perception of the retail food environment, including food availability and adequacy. Questions include what they usually buy at the retail store, how much they spend (on average) on those food items, where else do they usually go to buy food, how has COVID-19 affected how much food they buy at the retail store, reason for shopping at the particular store, the importance of specific factors in influencing food purchase and a question on customer satisfaction on the overall shopping experience at the particular retailer.

Prior to data collection the research teams will (i) conduct forward and backward translation of the questionnaire from English into the relevant local language(s), and (ii) pretest the questionnaire with local participants in each study country, to ensure the suitability of wording, terms used, sequencing of questions, interview time, and other issues likely to affect the accuracy and completeness of response. Data collection will be completed at different times of the day (morning, afternoon, evening) on all seven days of the week. Data will be collected across a range of time periods to accommodate individual shopping patterns [53]. Trained enumerators will conduct the consumer intercept survey on-the-spot whenever possible. If that is not possible (due to time/space constraints or COVID-19 restrictions), then the enumerator will obtain contact details of consenting respondents, and conduct online or telephone interviews at an appointed time. Research participants may be reimbursed for costs directly incurred during the data collection, such as travel costs, and compensated for their inconvenience and time spent. However, these reimbursement and compensation must be reviewed and approved by the local research ethics committee prior to participant recruitment.

##### Data Analysis

SPSS version 25 (IBM, New York, NY, USA) will be used for data analysis. Descriptive statistics will be used to characterize consumer purchasing behavior and perception of the retail food environment. Mean and standard deviation (SD) of each variable will be presented. Both bivariate and multivariate statistics will be used to examine the relationships between sociodemographic factors and the type of food retail and food products purchased by consumers. For bivariate analysis, cross-tabulation along with Fisher’s exact test will be conducted. Results of the multiple regression analysis will be estimated using maximum likelihood estimation.

#### 2.3.2. Assessment of the Consumer Retail Environment

##### Study Design and Setting

The researchers will expand on the Monitoring Availability, Placement and Promotion—Supermarkets (MAPP-S) audit tool [54], and adapt it for each study country. The MAPP-S instrument was developed in Australia, and is based on the validated INFORMAS food retail module used in New Zealand [55]. MAPP-S will be used to assess the in-store availability, placement, and promotion of healthy and unhealthy foods and beverages within different consumer food environments (i.e., within retail food outlets) using a validated indicator.

##### Data Collection

Data will be collected at the selected retail food outlets based on the findings of Phase One. This will involve measurement of the floor length and height/width allocated to particular products, a count of shelves allocated to various products, categorization of the location of various healthy and unhealthy products (e.g., end-of-aisle, checkout, island bin displays) and the type of price promotion used in these areas. The healthfulness of products was adapted from the Australian Dietary Guidelines [56], as all four countries in SEA do not have a specific criteria for the classification of healthy and unhealthy food. The food categories were then compared with each country’s Dietary Guideline [57,58,59,60], WHO Nutrient Profile Model for the Western Pacific Region [61], WHO Nutrient Profile Model for South East Asia region [62] and also from an article similar to the study, but conducted in South Africa [63]. Trained enumerators will conduct the audit assessment. Inter-rater reliability will be assessed based on two enumerators’ audit in assessing each category of retailer on the same day.

The researchers will review the MAPP-S-validated indicators by study country context, and conduct a pretest of the tool. Written approval will be sought from the retailer (e.g., store manager and/or head office) prior to data collection.

##### Data Analysis

Descriptive analysis will be used to calculate the mean value of each outcome variable, e.g., in-store availability, placement, and promotion of healthy and unhealthy foods and beverages. The mean values of these outcome variables will then be analyzed across different types of retailers in each study country by pairwise comparison. In addition, several multivariable linear regression models will be used to explore factors, which may have a statistically significant effect on the outcome variables. The analysis should help to explain how in-store availability, placement, and promotion of healthy and unhealthy foods and beverages vary across retailers.

### 2.4. Phase Three: Retailer Decision Making

The aim of this phase is to understand the factors influencing a food retailer’s decisions relevant to stocking policy, their perception and attitude toward food retail policies, and opportunities that will facilitate the supply and promotion of healthy and unhealthy foods through food retailing in each study country. Food retailers, as marketing firms, operate in bilateral contingencies [64,65]. Their behavior may be reinforced or disincentivized by consumer behavior through products and services while consumer behavior may also be reinforced or disincentivized by a retailer’s decisions [66].

#### 2.4.1. Study Design, Setting, and Population

This research will use an embedded mixed-method approach in which the quantitative data play a secondary role to the qualitative data [67]. The qualitative data collection phase will utilize semi-structured in-depth interviews with representatives of the different types of retailers (based on the findings in Phase One and similar to those in Phase Two). The interview will explore the following topics: (1) source of food being sold; (2) perspectives regarding producers and intermediaries in relation to health; (3) various food environment variables in the retail industry which facilitate or hamper the supply of healthy/unhealthy food through food retail, and which factors influence a retailer’s decision to supplying healthy/unhealthy food; (4) perceived effect of disruptions (e.g., an epidemic, typhoon, earthquake) on the supply of healthy/unhealthy food; (5) factors that impede or enhance response capacity to food supply disruptions; (6) perceptions of customer preferences.

In each country, at least 15 candidates for interviews (i.e., food retailers at a convenience store, supermarket, hypermarket) will be recruited through telephone or email. Eligible respondents should have the authority in making procurement, retail, and product placement decisions, and should have at least one year’s experience in his/her position at the retail food outlet.

#### 2.4.2. Data Collection

Open-ended interview questions (with prompts) will be used to explore the food retailer’s business strategies and their relationship with suppliers. The interview guide will probe emergent themes (food environment outcomes, retailer decision variables, availability/quality/acceptability/reliability of product, store footfall, customer level of satisfaction with a strategy for promoting ‘healthy food’, promos/branding, networks, supply chain resilience, etc.) and their interrelationships. Quantitative data will include retail food store characteristics (e.g., years in operation, type of ownership, mean store size, store footfall) and demographic information of the retailers. Before data collection, the interview guide will be pretested to validate the questions.

Interviews will be audio-recorded, and interviewees will be assured of anonymity of response. Written or oral informed consent will also be obtained from the interviewees.

#### 2.4.3. Data Analysis

Audio recordings will be transcribed non-verbatim (without distracting elements such as filler speech, false-starts, and idiosyncrasies) into a Microsoft Word file [68]. Member checking will then be conducted by providing interviewees with the opportunity to feed back or add to the interview content. The qualitative data will be analyzed using the following six steps of thematic analysis [69]: (1) familiarization with the data; (2) generation of initial codes to describe the content; (3) searching for themes or patterns; (4) review of themes; (5) definition of themes; and (6) production of the report of the thematic analysis. Through this process, themes, linkages, and explanations will be identified, and both deductive and inductive approaches will be used in the analysis. Coding will be completed by at least two researchers. Quantitative data will be analyzed using descriptive statistics.

The outputs from Phase Three will identify and provide a better understanding of factors influencing food retailer decisions, and their perception and attitude toward food retail policies and opportunities that could facilitate the supply of healthy food through product retailing in each study country. The outputs from this phase, together with the outputs from Phase Two, will inform the development of an interview guide for use in Phase Four.

### 2.5. Phase Four: Policy Landscape

The aim of this phase is to identify key opportunities for policy to improve the retail food environment, increase the availability of affordable healthier food in each study country, and decrease the availability of unhealthy food.

#### 2.5.1. Study Design, Setting and Population

This phase will use a policy space analysis approach [70,71] to analyze the policy landscape relevant to food retail in each country, including an analysis of policy documents and key informant interviews. Policy documents relevant to food retail will be identified through Internet search and direct overtures to representatives of national and local government agencies.

The interview guide will be informed by the points of leverage identified in previous phases, and will be used to examine how policy could incentivize positive (healthy) changes in the retail food environment. The final number of key informant interviews will be based on theoretical data saturation, i.e., the point at which no new themes or information emerge, and content becomes redundant. Following established methods for case study research [72], a likely sample size of 20–30 interviews per study country will be required. Therefore, in this phase, the research teams will initially conduct approximately 20 interviews with stakeholders and people involved in the policy process in each country. After the 20th interview, the research teams will continue to recruit and interview additional stakeholders until data saturation is achieved. All key informants will provide written informed consent prior to the interview.

#### 2.5.2. Data Collection

The key stakeholders involved in policy implementation will be identified using purposive sampling, implemented in two stages. The first stage will involve a search of policy documents through government websites and documents, NGO publications and websites, and major newspapers in each study country. In addition, the researchers will make direct overtures to representatives of key government ministries and non-government agencies (e.g., industry associations), and issue formal invitations to the heads of agencies to participate in the study. The second stage will involve snowball sampling to identify additional relevant stakeholders.

The content of the interview guide will be informed by findings of previous phases, and will focus on the following: (1) perceptions of the ‘problem’ of nutrition and diet-related NCDs; (2) understanding among policy actors of the potential role of retail food in improving diets; (3) political context; (4) stakeholder influence on current policy; (5) gender considerations. Prior to data collection, the interview guide will be pretested to validate the questions. Interviews will be conducted in person and audio-recorded.

#### 2.5.3. Data Analysis

In this phase, the researchers will first analyze relevant policies across sectors (e.g., industry, commerce, trade, urban planning) with a focus on current and potential incentives and disincentives relevant to healthy retail food, and use deductive thematic analysis based on the study framework. The following two criteria will be used to identify incentives: either (1) the concept of an incentive or facilitator is used, or (2) there is mention of something that may encourage or help to facilitate policymakers and/or government agencies to improve good governance for nutrition and increase healthy food retailing. Similarly, the following two criteria will be used to identify disincentives: either (1) the term ‘*disincentive*’ or ‘*barrier*’ is used, or (2) there is mention of something that deters the implementation of improvements to the nutrition and food retailing system. This phase will then draw on the interview data to analyze the policy context and stakeholders in order to better understand the political economy of retail food policy. Last, this phase will draw on the findings of previous phases to analyze how policy could address points of leverage for improving the healthiness of the retail food environment, and to provide policy recommendations.

After completion of Phase Four, a stakeholder meeting between government and non-government actors will be organized in each study country. The preliminary results from all phases and policy recommendations will be reported to the stakeholders. The meeting will expose a wide range of stakeholders to the results of the research, and solicit their professional opinion and critical assessment of the conclusions and the potential impact on the second opinion directive.

Following the in-country analysis, the researchers will examine the similarities and differences of the findings across study countries to identify any regional patterns in retail food policy, and distil any lessons at the regional level.

## 3. Discussion

To the best of the authors’ knowledge, this will be the first study to comprehensively analyze the retail food environment, consumer and retailer perspectives regarding the retail food environment, as well as existing policies influencing food retail in Indonesia, Malaysia, the Philippines, Thailand, and in SEA generally. A mixed-method approach to data collection will be applied, including quantitative (surveys) and qualitative (in-depth interviews) methods. Although Malaysia, the Philippines, and Thailand have previously conducted an assessment of their respective food environment as part of the INFORMAS Project, these assessments focused on the particular domain or setting of the food environment. Malaysia and the Philippines conducted their assessment of the food-promotion domain, while Thailand conducted an assessment of the food-retail domain only in the school setting [73]. There is no documentation of such an assessment of the food environment in Indonesia.

The proposed research is a collaboration of researchers who are already engaged in nutrition/food environment policy in each study country. The research is also designed so that there is ample opportunity for two-way engagement with policy actors in each study country.

This study will strategically engage stakeholders throughout the research to ensure that the design is sensitive to the local context and creates opportunities for policymakers and other stakeholders to be informed of and to apply the research findings. The research teams will convene open public forums for policy stakeholders (government and non-government) at the beginning and end of the study. Stakeholder consultations involving design and policy relevance have been completed. There will be an end-of-project forum to present final results of all four study countries. The researchers will also conduct one-on-one consultations mid-project in order to keep key stakeholders from government, civil society, academia, and the private sector engaged.

The researchers will disseminate key findings through policy briefs for every phase of the study, and produce final country reports for stakeholders.

The research teams in the four study countries will produce comprehensive case studies, and collect the data in such a way as to permit direct, cross-country comparisons. The structure of the government varies in important ways among the four study countries. This is an advantage, however, since it will allow comparisons of the food retail policy environment across different facets of socio-demographic and economic dimensions.

### Strengths and Limitations of the Proposed Research

The SEAOFE study will be the first international comparative study that provides comprehensive evidence for policies to facilitate healthier retail food and, thereby, en-courage consumption of healthy food. The study will analyze consumer experience and retailer perception of the food environment, the in-store retail environment, and existing food policy context in a single, integrated, comprehensive design.

This study brings together what are usually discrete studies. Conducting them in an interconnected way, brings information on communities, food retail and policy together to collectively strengthen the study findings.

The study will also provide a multi-stakeholder engagement platform for policy dialogue to discuss how to collaboratively address the growing burden of malnutrition through improving the retail food environment in the study countries and in SEA generally.

The protocol has been designed to allow each study country to adapt the tools and processes to fit the national context, and the protocol can also be applied in other countries with a similar context. In this way, the research has the potential to contribute to the collection of regional and global evidence of the food environment in low- and middle-income countries. That said, the proposed research is limited to the retail-ers around urban poor areas, with detailed data on only selected retail sectors in those areas in each study country. Thus, many forms of informal retail are not included. Other limitations may relate to sampling and recall bias of survey participants. Nonetheless, a broad understanding of the retail food environment in pre-COVID-19 and pandemic eras will be obtained from the literature review.

## 4. Conclusions

The SEAOFE study will generate evidence on consumer and retailer experience of the food environment, and the existing food policy context (including barriers and opportunities for policy change) in the SEA region.

The four study countries differ across economic and socio-demographic contexts. Therefore, the country case examples provide an opportunity to learn from each other’s progress and challenges, and will aid in identifying areas for continued work.

In this way, the research has the potential to contribute to the regional and global knowledge base on the food environment in low- and middle-income countries, and to improve understanding of the barriers and facilitators to policy change.

Any new evidence of food environment interactions, together with the detailed analysis of policy content and context will be used to identify specific opportunities for policy change that would facilitate consumption of healthy food through improving the external food environment in each of the four study countries and in SEA generally. The research will also inform key decision-makers directly through dissemination of policy briefs and stakeholder engagement.

## Data Availability

The datasets generated during the research will not be publicly available while the study is still ongoing. Data will be available from the corresponding author based on reasonable request once the study is completed. Data will be stored securely in a dedicated office within the Universiti Kebangsaan Malaysia. All electronic information will be backed up on an external hard drive. Data generated and/or analyzed during the study will be included in manuscripts to be submitted for publication in peer-reviewed journals.
